# Long non-coding RNA CCAL/miR-149/FOXM1 axis promotes metastasis in gastric cancer

**DOI:** 10.1038/s41419-018-0969-z

**Published:** 2018-09-24

**Authors:** Xi Luo, Gui-Hua Wang, Zhao-Lian Bian, Xi-Wen Li, Bing-Ying Zhu, Chun-Jing Jin, Shao-Qing Ju

**Affiliations:** 1 Laboratory Medicine Center, Affiliated Hospital of Nantong University, No.20 Xisi Road, 226001 Nantong, Jiangsu Province China; 2 Department of Clinical Laboratory, The Third People’s Hospital of Nantong, No.60 Middle Qingnian Road, 226006 Nantong, Jiangsu Province China; 3 Nantong Institute of Liver Diseases, The Third People’s Hospital of Nantong, No.60 Middle Qingnian Road, 226006 Nantong, Jiangsu Province China

## Abstract

Early evidence indicates that the long non-coding RNA CCAL plays a critical role in cancer progression and metastasis. However, the overall biological role and clinical significance of CCAL in gastric tumourigenesis and progression remain largely unknown. We observed that CCAL was upregulated in gastric cancer tissues and was associated with the tumour-node-metastasis stage. Functional experiments showed that CCAL promoted gastric cancer cell proliferation and metastasis in vitro and in vivo. Luciferase reporter assay indicated that CCAL directly bind to miR-149. Moreover, knockdown of CCAL significantly reduced the expression of FOXM1, a direct target of miR-149. We also showed that FOXM1 suppression by miR-149 could be partially rescued by CCAL overexpression. In addition, we identified a negative correlation between the mRNA expression of CCAL and miR-149 in gastric cancer tissues. Furthermore, we observed a negative correlation between the expression of miR-149 and FOXM1 and a positive correlation between CCAL and FOXM1 levels. These results demonstrated that the CCAL/miR-149/FOXM1 axis functions as a key regulator in gastric cancer metastasis and CCAL potentially represents a biomarker for diagnosis and potential target for therapy in the future.

## Introduction

Gastric cancer (GC) is the third leading cause of cancer-related death worldwide and the most common gastrointestinal malignancy in East Asia and Latin America^1–3^. Although the incidence and mortality rates have generally declined and surgical techniques have substantially improved during the past decades, the recurrence or metastasis rate of GC remains high, and the 5-year survival rate remains low^4^. The pathogenesis of GC is very complex and poorly understood. Genetic aspects, *H. pylori* infection, unhealthy eating habits and smoking may all lead to GC development and progression^5,6^. Thus, due to heterogeneity, the GC recurrence rate is relatively high and is not helpful for improving the quality of life of patients with metastasis to re-resection^7^. Therefore, exploring the underlying molecular mechanism of tumourigenesis and metastasis is critical for treating and monitoring GC.

Long non-coding RNAs (lncRNAs) is a class of non-coding RNAs which is longer than 200 nt and lack of protein-coding ability. In the past, lncRNAs were considered “trash RNA” without arousing much attention. In recent years, a massive amount of evidence has revealed that lncRNAs are involved in various cancer cell biological processes, such as cell growth, cell cycle distribution and cell metastasis^8,9^.

Accumulating evidence has demonstrated that many lncRNAs are dysregulated in GC, particularly HOXA11-AS, GClnc1, BC032469 and GAPLINC. These lncRNAs function as tumour oncogenes or suppressors, depending on the circumstances. HOXA11-AS promotes GC metastasis by regulating β-catenin and KLF2^10^. GClnc1 is highly expressed in GC and modulates the interaction of the WDR5 and KAT2A complex^11^. Lu et al. found that BC032469 promoted cell proliferation and upregulated hTERT expression by completely sponging miR-1207 in GC^12^. Hu et al. revealed that GAPLINC regulated CD44 as a molecular decoy for miR-211-3p^13^. Therefore, it is necessary to further investigate GC-associated lncRNAs.

Ma et al. identified a new functional lncRNA in colorectal cancer (CRC) and named it colorectal cancer-associated lncRNA (CCAL)^14^. This author showed that CCAL promoted CRC cell proliferation and migration by targeting AP-2α, which in turn activated the Wnt/β-catenin pathway. Liu et al. found similar results in hepatocellular carcinoma^15^. Zhou et al. demonstrated that CCAL acted as an oncogene in osteosarcoma (OS) and might be an independent prognostic factor for OS patients^16^. Ye et al. demonstrated that CCAL promoted papillary thyroid cancer development and progression by activation of the NOTCH1 pathway^17^. Shan et al. reported that CCAL promoted gastric cancer cell proliferation and migration in a Myc-dependent manner^18^. However, whether CCAL exerts its oncogenic effect on GC through other mechanisms remains unclear. In the present study, the biological roles of CCAL in GC development were explored in vitro and in vivo. More importantly, we found that CCAL could bind to miR-149, suppress the translation of Fork head box M1 (FOXM1), and subsequently promote metastasis in gastric cancer. The CCAL/miR-149/FOXM1 axis may act as a potential target for GC therapy.

## Results

### CCAL is upregulated in human GC tissues and cells

**Fig. 1:**
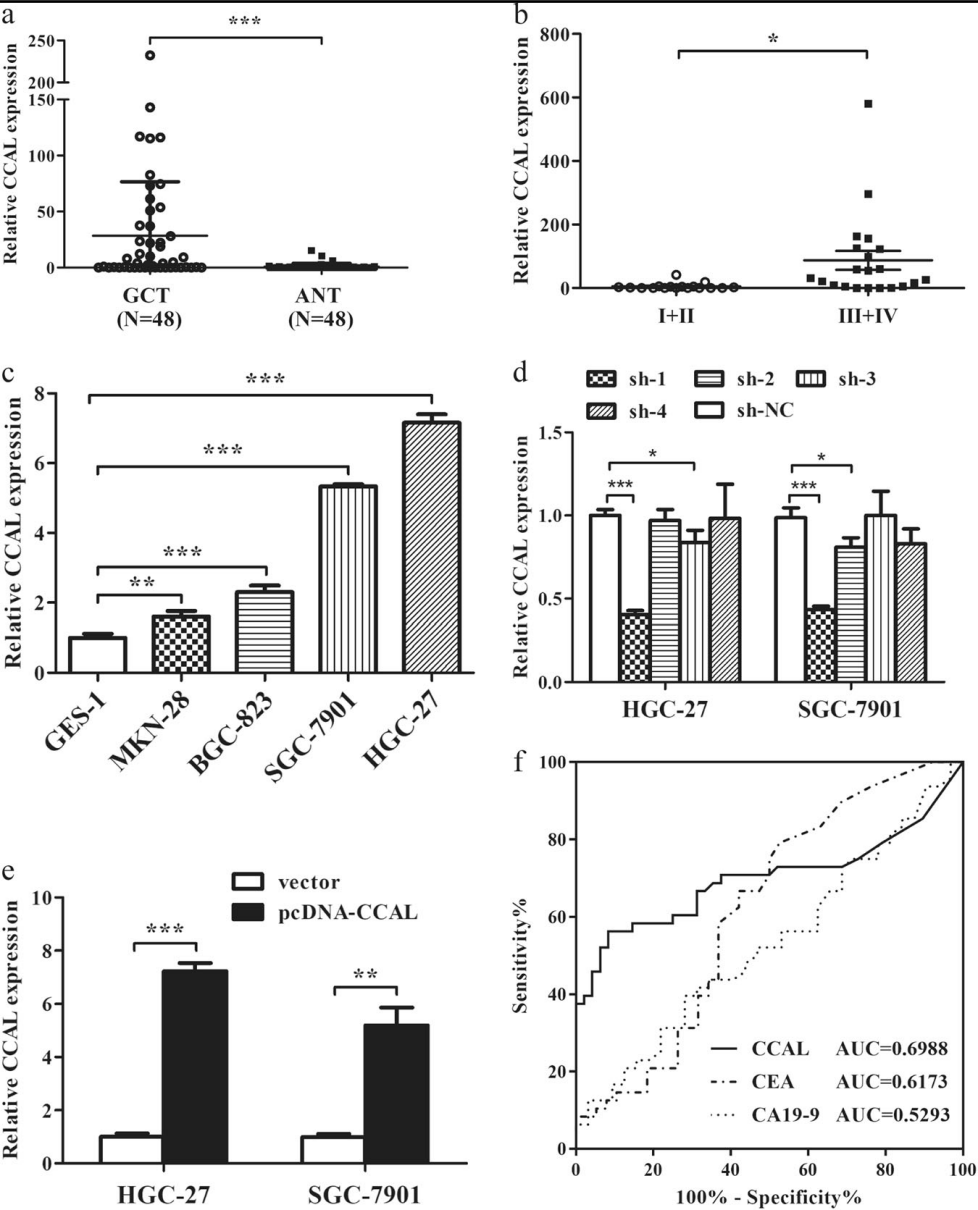
The expression of CCAL in gastric cancer tissues and cell lines. **a** Relative CCAL expression in gastric cancer and paired adjacent normal tissues were detected by quantitative real-time polymerase chain reaction (qRT-PCR) (*N* = 48). **b** Relative CCAL expression in the gastric cancer patients for stage I + II and stage III + IV. **c** Relative CCAL expression in gastric cancer cell lines (BGC-823, HGC-27, MKN-28 and SGC-7901) compared to normal gastric epithelial cell line GES-1. **d** Relative CCAL expression level in gastric cancer cells after transfected with shRNA or sh-NC. **e** Relative CCAL expression level in gastric cancer cells after transfected with pcDNA-CCAL or vector. Data was presented with mean ± SD, the error bars represent the SD obtained from three independent experiments. **f** ROC curve for prediction of gastric cancer using CCAL expression level. The AUC was 0.6988, ROC for CEA and CA19-9 were also been showed. *P < 0.05, **P < 0.01, ***P < 0.001

To investigate the expression and clinical significance of CCAL in GC, we first measured the mRNA levels of CCAL in 48 pairs of GC tissues and the paired adjacent normal tissues by qRT-PCR, normalizing to 18 S rRNA. The CCAL expression level was significantly elevated in the tumour tissues (28.51 ± 48.11) compared with that in the adjacent normal tissues (0.96 ± 2.76) (Fig. 1a) (*P* < 0.001). The CCAL level in stage I + II patients was lower than stage III + IV patients (Fig. 1b) (*P* = 0.0178).

Next, we examined the correlation between the CCAL expression level and clinical features in GC patients. High levels of CCAL were correlated with Lauren type (*P* = 0.017), AJCC clinical stage (*P* = 0.008), and advanced TNM stage (*P* < 0.001) (Table 1). However, CCAL expression was not associated with the other clinicopathological features, such as patient gender, age, tumour size, location, differentiation, smoking, drinking alcohol, *H. pylori* infection, serum CEA, CA19-9 and lymph nodes in our study.

**Table 1:** Correlation of the expression of CCAL with clinical features in gastric cancer

Characteristics	CCAL expression		*P*-value
	Low group (*N* = 24)	High group (*N* = 24)	
Age (y)			0.186
<60	7	9	
≥60	17	15	
Gender			0.768
Male	15	14	
Female	9	10	
Diameter (cm)			0.391
<5	12	15	
≥5	12	9	
Location			0.422
Proximal	5	8	
Middle	16	15	
Distal	3	1	
Differentiation			0.265
Poor	13	17	
Moderately	9	4	
Highly	2	3	
Lauren type			*0.017
Intestinal	19	11	
Diffuse or mixed	5	13	
AJCC clinical stage			*0.008
I	14	5	
II + III	10	19	
Drinking alcohol			0.763
No	8	9	
Yes	16	15	
Smoking			0.540
No	15	17	
Yes	9	7	
TNM stage			*<0.001
I + II	16	4	
III + IV	8	20	
Lymphatic metastasis			0.248
N0 + N1	12	8	
N2	4	9	
N3	8	7	
CEA			0.710
Negative	15	18	
Positive	9	6	
CA19-9			0.490
Negative	18	23	
Positive	6	1	
HP			0.365
Negative	14	17	
Positive	10	7	

Then, the expression levels of CCAL in the GC cell lines were detected by qRT-PCR, and we found that the expression levels of CCAL in GC cell lines were significantly higher than that in the normal gastric epithelium cell line GES-1 (Fig. 1c). HGC-27 and SGC-7901 cells expressed relatively higher levels of CCAL, whereas BGC-823 and MKN-28 cells expressed relatively lower levels of CCAL; therefore, HGC-27 and SGC-7901 cells were chosen for further study. To avoid an off-target effect, we designed four candidate shRNAs, and sh-1 had optimized interference efficiency. Relative CCAL expression in HGC-27 and SGC-7901 after knockdown or overexpression was detected by qRT-PCR (Fig. 1d, e).

### CCAL may be used as a potential biomarker in GC diagnosis

The ROC curve was determined to evaluate the sensitivity and specificity of the diagnostic value based on CCAL expression. Notably, CCAL displayed a considerable diagnostic significance, with an area under curve (AUC) of 0.6988, more powerful than CEA (AUC = 0.6173) and CA19-9 (AUC = 0.5293) (Fig. 1f), suggesting that CCAL could serve as a more valuable tumour marker for GC diagnosis.

### CCAL promotes cell proliferation, cell cycle progression and inhibits apoptosis of GC cells in vitro

**Fig. 2:**
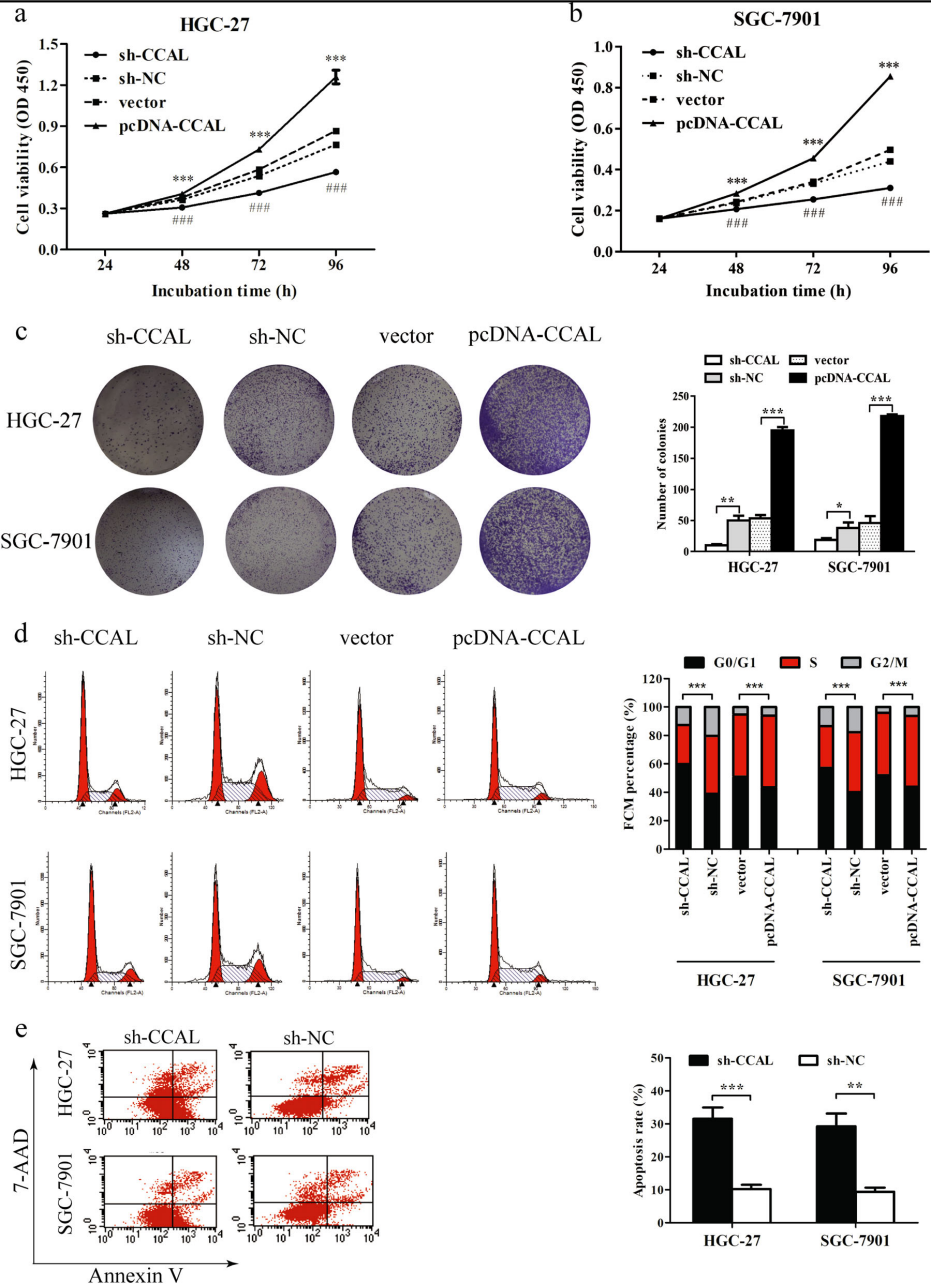
CCAL promotes proliferation, cycle progression and inhibits apoptosis of gastric cancer cells in vitro. **a**, **b** CCK-8 assays were used to determine the cell viability of gastric cancer cells transfected with sh-CCAL or pcDNA-CCAL with relative control. *pcDNA-CCAL; #sh-CCAL. **c** Colony formation assays were used to determine the cell colony formation ability of sh-CCAL or pcDNA-CCAL-transfected gastric cancer cells. The number of colonies was calculated and plotted on a histogram. **d**, **e** FACS analysis was used to determine the effect of sh-CCAL or pcDNA-CCAL on cell cycle and apoptosis. *P < 0.05, **P < 0.01, ***P < 0.001

CCK-8 assay revealed that cell growth was significantly impaired in HGC-27 and SGC-7901 cells transfected with sh-CCAL, while cell growth was accelerated in pcDNA-CCAL-transfected cells compared with respective controls (Fig. 2a, b) (*P* < 0.001). Similarly, the result of the colony formation assay revealed that the colony formation rate was decreased following the inhibition of CCAL in GC cells, whereas CCAL overexpression had the opposite effect (Fig. 2c).

We further analysed cell cycle distribution using flow cytometry in sh-CCAL-treated HGC-27 and SGC-7901 cells. In comparison with sh-NC-transfected cells, both sh-CCAL-transfected cell lines showed cell cycle arrest in G0/G1 phase 48 h after transfection, characterized by the presence of nearly 60% of cells in G0/G1 phase of the cell cycle and the presence of less than 30% of cells in S phase. In contrast, the proportion of cells in G0/G1 phase was significantly decreased in GC cells transfected with pcDNA-CCAL compared with those transfected with vector. Meanwhile, compared with the control group, the pcDNA-CCAL group displayed more cells in S phase (Fig. 2d). The results showed that CCAL promoted cell cycle progression in these two GC cell lines.

We performed flow cytometry analysis to determine whether apoptosis was a contributing factor to cell growth inhibition. The data showed that the percentage of apoptotic cells was significantly higher in GC cells transfected with sh-CCAL than in the sh-NC group, indicating that knockdown of CCAL induced apoptosis in GC cells (Fig. 2e).

### Knockdown of CCAL inhibits the invasion and migration of GC cells in vitro

**Fig. 3:**
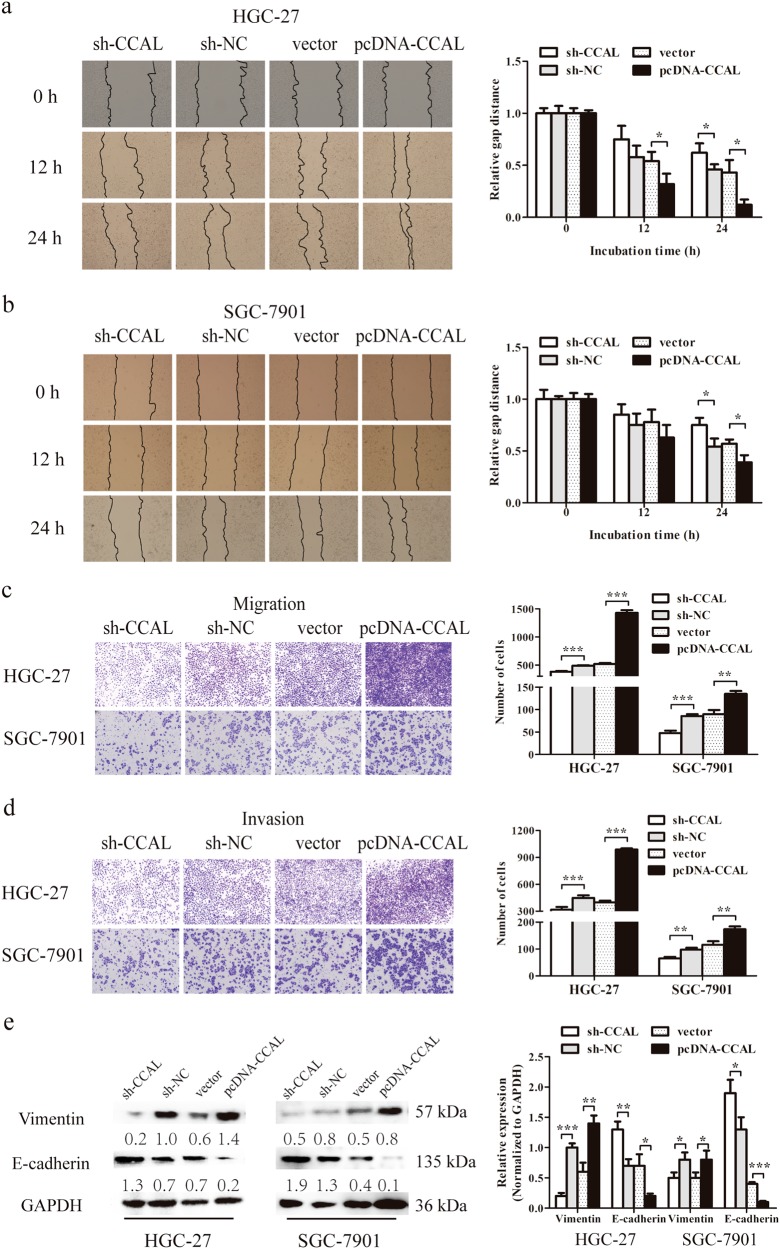
CCAL promotes cell migration and invasion in gastric cancer cells. **a**, **b** Wound-healing assay were used to investigate the horizontal migration ability with CCAL knockdown or overexpression in gastric cancer cells, and relative gap distance was calculated and plotted on a histogram. **c**, **d** Migration and invasion assays were used to investigate the vertical migration and invasion abilities with CCAL knockdown or overexpression in gastric cancer cells, and the number of cells was calculated and plotted on a histogram. **e** Western blot was used to analyze the expression of metastasis-related proteins with CCAL knockdown or overexpression in gastric cancer cells. Data from Western Blot assay has been represented as a quantification graph normalized to the levels of GAPDH together with the statistical tests. *P < 0.05, **P < 0.01, ***P < 0.001

To evaluate whether CCAL contributes to the metastasis of GC, we examined the effect of CCAL on the migration and invasion abilities of HGC-27 and SGC-7901 cells. HGC-27 and SGC-7901 cells transfected with pcDNA-CCAL displayed a notably faster recovery than controls; conversely, GC cells with knocked down CCAL showed a slower recovery than controls (Fig. 3a for HGC-27, b for SGC-7901). Transwell assays revealed that the migration and invasion abilities of the GC cells were dramatically impaired following the downregulation of CCAL. Conversely, CCAL overexpression increased the migration and invasive abilities of the GC cells (Fig. 3c for migration, d for invasion).

To investigate the proteins involved in cell metastasis under the regulation of CCAL, the expression levels of some oncogene or tumour-suppressor-related proteins related to tumour metastasis in sh-CCAL GC cells were examined. The expression of vimentin, an important protein related to tumour metastasis, was downregulated, whereas that of E-cadherin was markedly elevated in sh-CCAL GC cells, suggesting that the change in these proteins may be involved in sh-CCAL-mediated malignant progression. The opposite result was also noted in GC cells in which CCAL expression was overexpressed using pcDNA-CCAL (Fig. 3e).

### CCAL promotes GC cell proliferation and metastasis in vivo

**Fig. 4:**
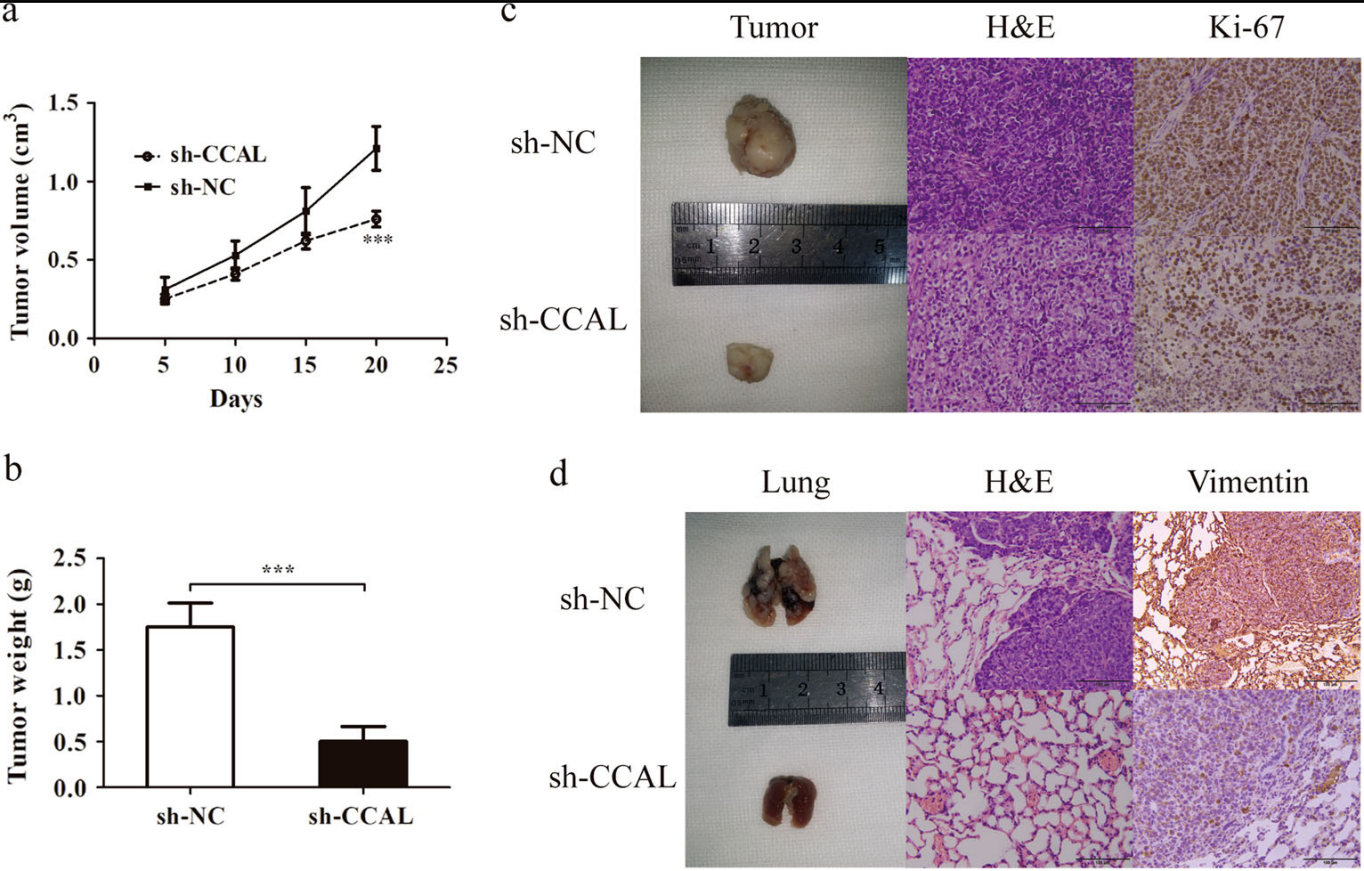
Knockdown of CCAL inhibits gastric cancer cell tumour growth and metastasis in vivo. **a** Tumour volume was measured every 5 days. **b** The mean weight of sh-CCAL group-derived xenograft tumours was also significantly less than sh-NC group-derived xenograft tumours. **c** Representative images of the xenograft tumours obtained from sh-NC or sh-CCAL group. Left, images of the xenograft tumours from the two group. Middle, images of H&E-stained tumour tissues. Right, immunohistochemical images showing the intensity of Ki-67 expression. **d** Representative images of the lungs obtained from sh-NC or sh-CCAL group. Left, images of the lungs from the two group. Middle, images of HE-stained lung tissues. Right, immunohistochemical images showing the intensity of vimentin expression. ***P < 0.001

To determine whether CCAL affected tumourigenesis, we injected HGC-27 cells transfected with either sh-NC or sh-CCAL into female nude mice. For the in vivo tumour growth assay, cells was subcutaneously injected into the left flank area of each mouse. Tumour growth in sh-CCAL group was significantly inhibited as demonstrated by decreased mean volumes and weights as well as slower tumour growth rates compared with those of tumours in the sh-NC group (Fig. 4a, b). In addition, H&E staining revealed that cells formed from the sh-CCAL group significantly reduced tumourigenesis than cells formed from the sh-NC group. Immunohistochemical staining of tumour tissues indicated a decrease in Ki-67 in the sh-CCAL group compared with that in the sh-NC group (Fig. 4c). To evaluate the effect of CCAL expression on GC metastasis, we injected transfected cells into nude mice via the tail vein. After 6 weeks, the mice were sacrificed, and lungs were obtained for further analysis. The sh-CCAL group showed a lower degree of metastatic nodules in lungs than the sh-NC group. H&E staining of lung sections further confirmed this difference. In addition, the expression level of vimentin in the metastatic tumour nodules that of the sh-CCAL group was reduced as detected by IHC (Fig. 4d).

### CCAL negatively regulates miR-149

**Fig. 5:**
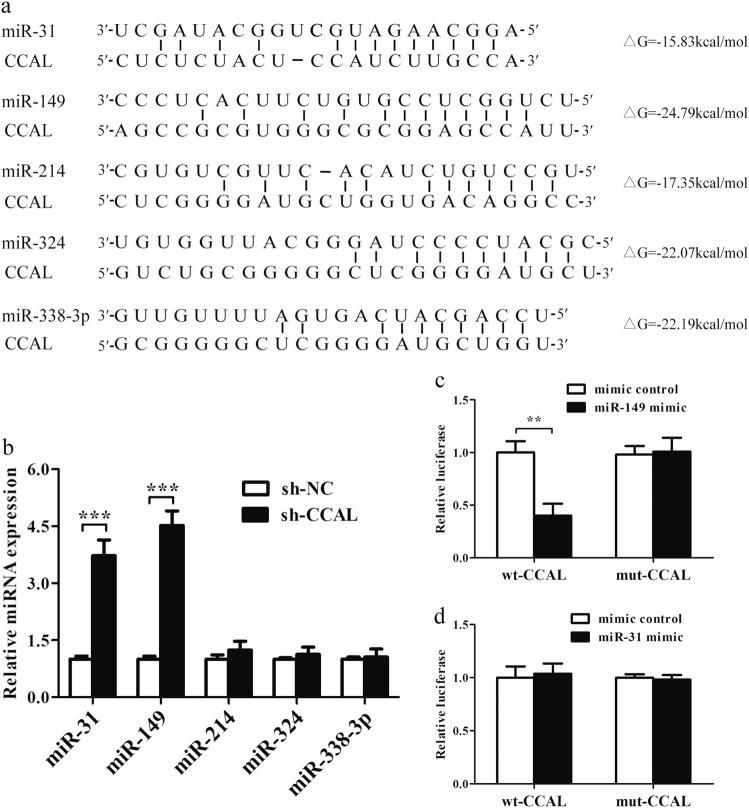
MiR-149 is a direct target of CCAL. **a** The predicted positions of candidate miRNAs binding sites on the CCAL transcript. **b** QRT-PCR for miRNA levels in SGC-7901 cells after transfected with sh-CCAL. Luciferase activity in 293 T cells co-transfected with miR-149 (**c**) or miR-31 (**d**) mimics and the luciferase reporters (the mutant type CCAL) or control (the wild type CCAL). Renilla luciferase activity was measured and normalized to firefly luciferase. The experiment was repeated at least three times, and data are presented as the mean ± SD (Two-sided Student’s *t*-test). **P < 0.01, ***P < 0.001

To further study the potential mechanism of CCAL involved in the development of GC, we used the bioinformatics software miRanda and TargetScan to identify the miRNAs that could bind to CCAL. The binding sites of CCAL and potential target miRNAs, including miR-31, -149, -214, -324 and -338-3p, were shown (Fig. 5a). The expression of the above miRNAs in SGC-7901 cells after transfection with sh-CCAL was detected by qRT-PCR. After knocking down CCAL, the expression of miR-31 and miR-149 was significantly elevated, while the other three miRNAs had no obvious changes in expression (Fig. 5b) (*P* < 0.001). Introduction of the miR-149 mimic significantly reduced the luciferase activity from the wt-CCAL compared with the negative control but did not affect the luciferase activity from the mut-CCAL (Fig. 5c) (*P* < 0.01). Conversely, compared with the negative control, miR-31 had no inhibitory effect on the luciferase activity from the wt-CCAL (Fig. 5d).

**Fig. 6:**
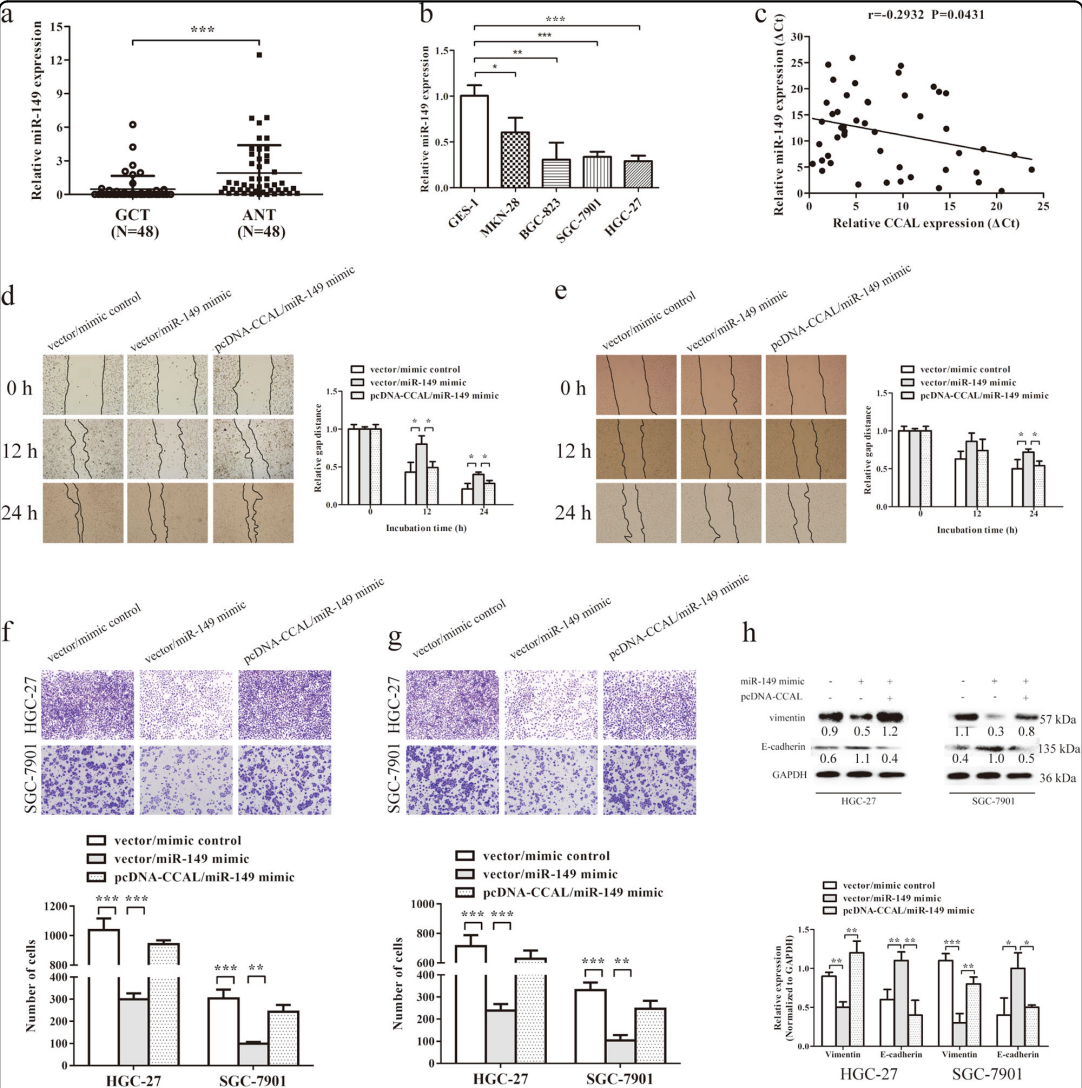
CCAL negatively regulates miR-149. **a** Relative miR-149 expression level in gastric cancer and adjacent normal tissues was detected by qRT-PCR (*N* = 48). **b** Relative miR-149 expression in gastric cancer cell lines normalized to GES-1. **c** The linear correlation between the expression levels of CCAL and miR-149 in gastric cancer tissues (*r* = −0.2932) (*P* = 0.0431). Wound-healing assays were conducted in HGC-27 (**d**) and SGC-7901 (**e**) cells transfected with miR-149 mimic or co-transfected with miR-149 mimic and pcDNA-CCAL, and relative gap distance was calculated and plotted on a histogram. Migration (**f**)and invasion (**g**) assays were used to investigate the vertical migration and invasion ability in gastric cancer cells, and the number of cells was calculated and plotted on a histogram. **h** Western blot was used to analyze the expression of metastasis-related proteins in gastric cancer cells. *P < 0.05, **P < 0.001, ***P < 0.001

Next, we used qRT-PCR to measure the mRNA levels of miR-149 in 48 pairs of GC tissues and the corresponding adjacent normal tissues, normalized to 18 S rRNA. The results indicated that the miR-149 expression level was significantly lower in tumour tissues (0.47 ± 1.19) than in the adjacent normal tissues (1.91 ± 2.49) (Fig. 6a) (*P* < 0.001). QRT–PCR analyses also revealed that GC cell lines had lower levels of miR-149 expression than GES-1 cells, while SGC-7901 and HGC-27 cells expressed relatively deeper levels (Fig. 6b). We also observed a negative relationship between the expression levels of CCAL and miR-149 in the same GC tissue samples (*r* = −0.2932) (Fig. 6c) (*P* = 0.0431).

To investigate the biological role of miR-149 in gastric cancer cells, we performed wound-healing assays. The overexpression of miR-149 could largely impair the migration ability of GC cells, which could be partly restored by pcDNA-CCAL (Fig. 6d for HGC-27, e for SGC-7901). Similar results were indicated by using Transwell assays (Fig. 6f for migration, g for invasion). Western blot was subsequently performed to investigate the protein expression. The expression of vimentin was downregulated and that of E-cadherin was elevated in miR-149 overexpressing GC cells. When pcDNA-CCAL was added, the expression of vimentin was increased and that of E-cadherin was decreased at the protein level (Fig. 6h). Taken together, these data clearly demonstrated that CCAL promoted GC cell migration and invasion by negatively regulating miR-149.

### The CCAL /miR-149/FOXM1 axis promotes metastasis of GC

We further investigated the downstream targets of miR-149 that function in GC cells. Based on two databases (Pictarget and TargetScan), Fork head box M1 (FOXM1) was selected as a putative target of miR-149, which was also reported in non-small cell lung cancer (NSCLC)^19^. FOXM1 is a transcription factor of the Fork head box protein superfamily, and this gene, located on human chr 12, is ~23 kb in length.

**Fig. 7:**
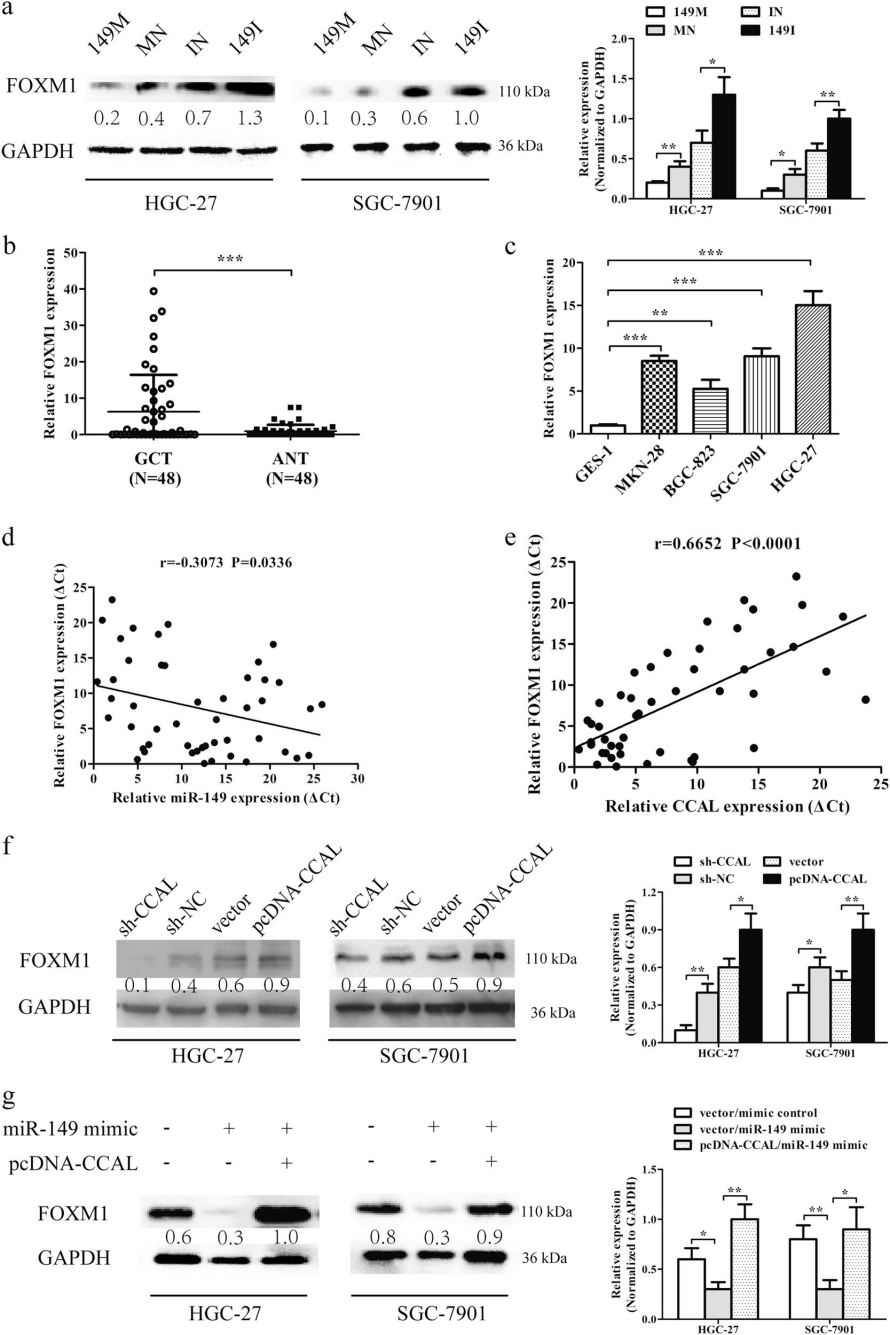
The CCAL /miR-149/FOXM1 axis promotes metastasis of gastric cancer. **a** MiR-149 negatively regulates FOXM1 in gastric cancer cells. 149 M, MN, IN, 149I represent miR-149 mimic, mimic control, inhibitor control, miR-149 inhibitor, relatively. **b** Relative FOXM1 expression in gastric cancer tissues (*N* = 48). **c** Relative FOXM1 expression in gastric cancer cell lines normalized to GES-1. **d** The linear correlation between the expression levels of miR-149 and FOXM1 in gastric cancer tissues (*r* = −0.3073) (*P* = 0.0336). **e** The linear correlation between the expression levels of CCAL and FOXM1 in gastric cancer tissues (*r* = 0.6652) (*P* < 0.001). **f** CCAL positively regulates FOXM1 in gastric cancer cells. **g** Western blot assays were performed to test FOXM1 expression after HGC-27 and SGC-7901 cells were transfected with miR-149 mimic or co-transfected with miR-149 mimic and pcDNA-CCAL. Data from Western Blot assay has been represented as a quantification graph normalized to the levels of GAPDH together with the statistical tests. *P < 0.05, **P < 0.001, ***P < 0.001

Next, we analysed the changes in FOXM1 expression in GC cells after the ectopic overexpression or silencing of miR-149 and showed that the upregulation of miR-149 could significantly reduce the expression of FOXM1 at the protein level and that silencing of miR-149 could lead to the upregulated expression of FOXM1 at the protein level (Fig. 7a). These data suggest that FOXM1 may be a direct target of miR-149 in GC cells.

We found that FOXM1 had strong positive expression in GC tissues, and weak negative expression in normal tissues, consistent with the findings of previous studies^20,21^. Additionally, qRT-PCR was performed to measure the mRNA levels of FOXM1 in 48 pairs of GC tissues and the corresponding adjacent normal tissues. The results showed that the FOXM1 mRNA levels were higher in GC tissues than in normal gastric tissues (6.25 ± 10.14 vs 0.94 ± 1.75) (Fig. 7b) (*P* < 0.001). QRT–PCR analyses also revealed that GC cell lines had higher levels of FOXM1 expression than GES-1 cells (Fig. 7c). Then, we observed that the mRNA level of FOXM1 was negatively correlated with miR-149 (*r* = −0.3073) (Fig. 7d) (*P* = 0.0336) and positively correlated with CCAL in GC tissues (*r* = 0.6652) (Fig. 7e) (P < 0.001).

The concordant inverse correlation between CCAL and miR-149 and the negative relationship between miR-149 and FOXM1 indicated that the CCAL/miR-149/FOXM1 axis functions as an important player in GC cell invasion and migration.

Western blot was performed to investigate how CCAL/miR-149/FOXM1 posed impacts on GC cell metastasis. The result showed that the knockdown of CCAL resulted in a decrease in FOXM1 expression, while CCAL was overexpressed and FOXM1 expression was increased at the protein level (Fig. 7f). In addition, transfected miR-149 mimics downregulated FOXM1 expression, and addition of pcDNA-CCAL could restore the expression of FOXM1 (Fig. 7g).

## Discussion

In the present study, we revealed that the long non-coding RNA CCAL was significantly upregulated in gastric cancer tissues compared to that in adjacent normal tissues, consistent with a previous report^18^. This result implies that the high expression level of CCAL is significantly associated with Lauren type, AJCC clinical stage and advanced TNM stage. .

**Fig. 8:**
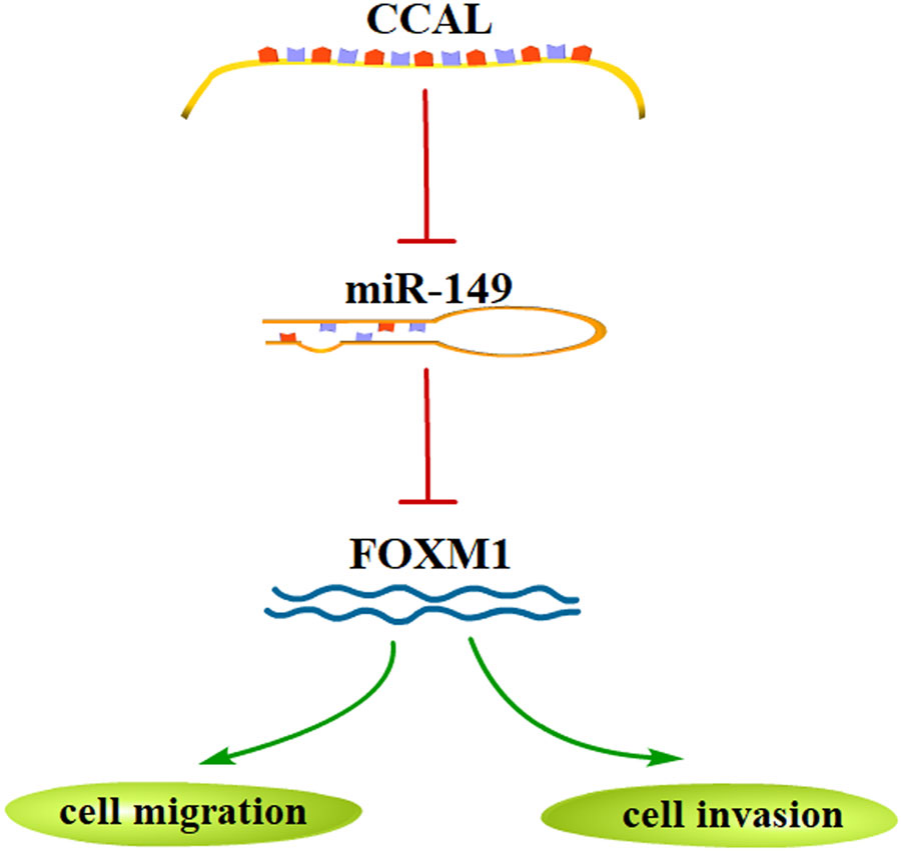
Model for CCAL-regulating FOXM1 by CCAL/miR-149/FOXM1 axis

Over the past decades, a number of protein-coding genes have been identified as valuable biomarkers for clinical diagnosis^22,23^. However, these biomarkers lack sufficient sensitivity and specificity. Additionally, a growing number of studies have demonstrated that non-coding RNAs, predominantly miRNAs, could serve as potential biomarkers of GC^24,25^. Considering that lncRNAs are more abundantly expressed in mammalian cells, it is plausible to speculate that lncRNAs, once regarded as “transcriptional noise”, may be potential diagnostic indicators in GC. Therefore, we used a ROC curve to evaluate the diagnostic utility these molecules. The results indicated that CCAL provides more powerful differential ability than CEA and CA19-9, suggesting that CCAL could serve as a promising tumour marker for GC diagnosis.

We identified the function of CCAL in GC cells by applying gain or loss of function approaches. Knockdown of CCAL significantly suppressed the proliferation and cell cycle progression and promoted apoptosis in GC cells. In addition, these data clearly demonstrated that the upregulation of full-length CCAL promoted the proliferative and metastatic behaviours of GC cells both in vitro and in vivo.

Although CCAL acts as an oncogenic lncRNA that promotes the tumourigenesis and progression of GC according to a previous study^18^, whether CCAL could function as miRNA sponges like many other lncRNAs located in the cytoplasm remains unknown. Therefore, bioinformatics analyses and luciferase reporter assays were performed to validate the regulation relationship between CCAL and its target miR-149. Previous studies have shown that miR-149 functioned as a tumour suppressor in human cancers^26,27^. However, the role of miR-149 in the metastasis of GC cells remains poorly understood. Metastasis, an important developmental step in cancer progression, refers to the process of cancer cells disseminating from the primary foci to a distant focus. This transition requires a series of complex processes, including cancer cells escape from the primary foci, enter and survive in lymphatic circulation, enter secondary cancer, form micro-metastatic cancer and induce angiogenesis, all of which results in metastatic cancer. Metastasis not only changes cell morphology but also induces cells to acquire essential new functions, such as migration and invasion.

Due to CCAL stimulated the metastasis of gastric cancer, we used Western blot to analyse the expression changes of metastasis-relative proteins in response to CCAL or miR-149 changes. Generally, the important hallmarks of EMT include the loss of E-cadherin and increased expression of vimentin. We observed that elevated CCAL could repress miR-149 expression through the EMT phenotype by upregulation of mesenchymal cell marker vimentin and downregulation of epithelial cell marker E-cadherin. The findings indicate that has a significant impact on miR-149 through the metastasis by mediating vimentin and E-cadherin in GC cells. As many lncRNAs function as miRNA sponges in the cytoplasm, binding with miRNA would increase the protein level of target genes. To investigate whether CCAL-induced reduction in miR-149 would result in the upregulation of its mRNA target in GC, we focused on the miR-149 target gene FOXM1 for further investigation. FOXM1, a crucial member of the Fork head family, plays an essential role in GC initiation, angiogenesis, proliferation and metastasis^28^. Recent studies have strongly suggested that FOXM1 is involved in angiogenesis, invasion and metastasis, indicating that FOXM1 may be an oncogene^29,30^. Based on the published articles, the expression of FOXM1 is highly expressed in almost all malignancies and is closely related to tumour invasion and metastasis^31^. Metastasis is an important feature of malignant tumours, and mainly contributes to tumour recurrence and death. Xu showed that miR-149 can directly inhibit FOXM1, thereby inhibiting the invasion and migration of colorectal cancer cells^27^. However, the relationship between miR-149 and FOXM1 in GC has not previously been reported. Here, we showed that FOXM1 expression could be regulated by not only miR-149 but also CCAL. We observed a positive correlation between the mRNA levels of FOXM1 and CCAL expression in GC tissues. An inverse correlation was observed between the mRNA levels of FOXM1 and miR-149 expression. Similar to the study by Xu et al., we confirmed that CCAL could rescue the miR-149-induced inhibition on FOXM1^27^. In conclusion, CCAL induced the activation of GC cell migration and invasion by competitively binding with miR-149 and upregulating FOXM1 (Fig. 8).

The present study is the first convincing report to demonstrate that CCAL partially participates in gastric cancer cell metastasis by functioning as a miRNA sponge. The results also imply that CCAL might be a potential diagnostic target for gastric cancer patients. Notably, we did not demonstrate which pathway of the CCAL/miR-149/FOXM1 axis participates in GC and whether the CCAL expression level in GC patients correlates with patient survival. Thus, further investigations of prognostic and mechanism data are needed.

## Materials and methods

### Clinical specimens

Between May 2014 and March 2017, a total of 48 fresh GC specimens and paired adjacent normal tissues were collected from GC patients with informed consent and frozen at −80 °C. None of the patients received any treatment before surgery. The diagnosis of all specimens was histopathologically confirmed by pathologists. The study was approved by the Ethics Committee of the Affiliated Hospital of Nantong University (Nantong, Jiangsu, China).

### Cell culture

Human GC cell lines, including BGC-823, HGC-27, MKN-28 and SGC-7901, and the human normal gastric epithelium cell line GES-1 were obtained from the Cell Bank of the Chinese Academy of Sciences (Shanghai, China). GC cells and GES-1 were cultured in RPMI-1640 (Corning, Manassas, Virginia, USA) with 10% foetal bovine serum (FBS) (Gibco, Grand Island, NY, USA) at 37 °C in a humidified incubator under 5% CO_2_ conditions.

### RNA extraction and qRT-PCR assays

Total RNA was extracted using a mirVana™ miRNA Isolation Kit 1561 (Thermo Fisher Scientific, Waltham, MA, USA). The RNA was reverse transcribed to cDNA using a RevertAid RT Reverse Transcription Kit (Thermo Fisher Scientific) according to the manufacturer’s instructions. The 18 S rRNA gene was synthesized by Thermo Fisher Scientific and used as an internal control. All qRT–PCR assays were performed with the FastStart Universal SYBR Green Master Mix (Roche, Mannheim, GER). The primers sequences are shown in Table 3. Bulge-loop^TM^ miRNA qRT-PCR Primer Sets specific for miRNA is designed by RiboBio (Guangzhou, China), as well as all the miRNA mimic and inhibitor.

**Table 2:** The sequences of shRNA for CCAL

sh-CCAL-1	Sense	5′-GATCCGTTTCCAGAACTGGCAGCCCTTCCTGTCAGAGGCTGCCAGTTCTGGAAACTTTTTG-3′
	Anti-sense	5′-AATTCAAAAAGTTTCCAGAACTGGCAGCCTCTGACAGGAAGGGCTGCCAGTTCTGGAAACG-3′
sh-CCAL-2	Sense	5′-CACCGCATCTTAGACTGGATCTTCATTCAAGAGATGAAGATCCAGTCTAAGATGCTTTTTTG-3′
	Anti-sense	5′-GATCCAAAAAAGCATCTTAGACTGGATCTTCATCTCTTGAATGAAGATCCAGTCTAAGATGC-3′
sh-CCAL-3	Sense	5′-CACCGGTGCCCAAAGGTTAACTTTCTTCAAGAGAGAAAGTTAACCTTTGGGCACCTTTTTTG-3′
	Anti-sense	5′-GATCCAAAAAAGGTGCCCAAAGGTTAACTTTCTCTCTTGAAGAAAGTTAACCTTTGGGCACC-3′
sh-CCAL-4	Sense	5′-CACCGAGACAAAGTCGGTCAACTCTTTCAAGAGAAGAGTTGACCGACTTTGTCTCTTTTTTG-3′
	Anti-sense	5′-GATCCAAAAAAGAGACAAAGTCGGTCAACTCTTCTCTTGAAAGAGTTGACCGACTTTGTCTC-3′
sh-NC	Sense	5′-GATCCGAAGCCAGATCCAGCTTCCCTTCCTGTCAGAGGAAGCTGGATCTGGCTTCTTTTTG-3′
	Anti-sense	5′-AATTCAAAAAGAAGCCAGATCCAGCTTCCTCTGACAGGAAGGGAAGCTGGATCTGGCTTCG-3′

### Plasmid construction and transfection

Sh-CCAL and pcDNA-CCAL (pcDNA 3.1) with respective controls (sh-NC and vector) were designed by GenePharma (Suzhou, China). All shRNA sequences are shown in Table 2. GC cells were cultured in 6-well plates to obtain 50–60% confluence and then transfected using Lipofectamine 3000 (Thermo Fisher Scientific) according to the manufacturer’s protocol.

**Table 3:** The sequences for qRT-PCR

18s rRNA	Forward	5′-GTAACCCGTTGAACCCCATT-3′
	Reverse	5′- CCATCCAATCGGTAGTAGCG-3′
CCAL	Forward	5′-AAGGGAGTTTTGTGCGGTGAGAA-3′
	Reverse	5′- TGTGCTGGCTTGTTTGGCTTTATT-3′
FOXM1	Forward	5′-GCTTGCCAGAGTCCTTTTTGC-3′
	Reverse	5′- CCACCTGAGTTCTCGTCAATGC-3′

### CCK-8 and colony formation assay

Cell viability was monitored using Cell Counting Kit-8 (CCK-8) (Beyotime, Shanghai, China). At 48 h after transfection, the transfected cells were seeded onto 96-well plates at a density of 1 × 10^3^ cells/well. Cell viability detection was measured at 2 h after addition of CCK-8 reagent every 24 h under OD450.

The proliferation and colony formation of transfected GC cells were measured by colony formation assay. At 48 h after transfection, the cells were seeded onto 6-well plates at a density of 400 cells/well and maintained for 14 days in medium containing 10% FBS to enable colony formation, and the medium was replaced every 3 days. The colonies were then fixed with methanol and stained with 0.05% crystal violet (Beyotime). Visible colonies were counted manually.

### Cell cycle and apoptosis analysis

GC cells were harvested at 48 h after transfection by trypsin and washed three times with cold phosphate-buffered saline (PBS). For the cell cycle assay, the cells were fixed in cold 70% alcohol overnight at 4 °C, washed and then stained with propidium iodide (PI) (Solarbio, Beijing, China) containing RNase A. After incubation in the dark at 4 °C for 30 min, the cells were analysed within 1 h. The percentages of various cell cycle phases (G0/G1, S and G2/M phases) were counted and compared. For the apoptosis assay, the cells were mixed with a double staining Annexin V and 7-AAD Apoptosis Detection Kit (BD Pharmingen, San Jose, CA, USA) in the dark at room temperature for 15 min. Both experiments were analysed by flow cytometry analysis (BD) according to the manufacturer’s protocol.

### Wound-healing assay

Wound-healing assay was used to investigate the migratory ability of the transfected cells. When cell confluence reached approximately 100%, old medium was removed. A 10-μl tip was used to make a vertical wound, and cells were then washed three times with PBS to remove cell fragments and continuously cultured. Images of cells migrating into the wound were photographed at 0, 12 and 24 h using a microscope (Olympus, Tokyo, Japan). The relative distance between the gaps was photographed and measured under an inverted microscope (Olympus).

### Migration and invasion assays

For the migration assay, the cells were suspended at a density of 2 × 10^5^ cells/ml. For the invasion assay, the upper chamber was pre-coated with diluted Matrigel (BD). Matrigel was coagulated after 5 h, and the cells were suspended in RPMI-1640 at a density of 5 × 10^5^ cells/ml. A 100-μl aliquot of the cell suspension was then seeded onto the upper chamber (Corning, 8 μm). To attract the cells, 0.5 ml RPMI-1640 with 20% FBS was added in the bottom chamber. After incubation (16 h for invasion and 24 h for migration), the cells that migrated through the membrane were stained with methanol and 0.05% crystal violet (Beyotime). The number of migrated or invasive cells was determined from five random fields by using an optical microscope (Olympus).

### Western blot

Protein was extracted by RIPA lysis buffer containing phosphatase inhibitor, separated by SDS-polyacrylamide gel electrophoresis (SDS-PAGE, Beyotime), and then transferred onto 0.45-μm polyvinylidene fluoride (PVDF) membranes incubated with specific primary antibodies. Enhanced chemiluminescence (ECL, Millipore) chromogenic substrate was used to quantify the signals and GAPDH antibody was used as the control. The antibodies against vimentin, E-cadherin, FOXM1 were purchased from Cell Signaling Technology (CST, Boston, MA, USA).

### Lentiviral infection

The HGC-27 cell suspension was seeded onto a six-well plate at 3 × 10^4^ cells/well and incubated until 20% confluence was reached. Two experimental groups were constructed: sh-CCAL, which was transfected with sh-CCAL green fluorescent protein (GFP) lentivirus, and sh-NC, which was transfected with empty GFP lentivirus. An appropriate amount of lentivirus was added according to the multiplicity of infection (MOI = 20). GFP-tagged gene expression was observed under a fluorescence microscope on the third day after transfection, and cells with transfection efficiency > 80% were selected for subsequent analyses.

### In vivo tumour growth and metastasis assay

Female BALB/c nude mice (aged 4–5 weeks) were purchased from the Shanghai Experimental Animal Center of the Chinese Academy of Sciences. Animal handling and experimental procedures were approved by the Animal Experimental Ethics Committee of Nantong University. HGC-27 cells infected with sh-CCAL or sh-NC were harvested from 6-well cell culture plates and diluted to a concentration of 1 × 10^7^ cells/ml in cold PBS.

For the in vivo tumour growth assay, a total of 200 μl of suspended cells was subcutaneously injected into the left flank area of each mouse. Tumour growth was examined every 5 days, and tumour volumes were calculated (0.5 × length × width^2^). After 6 weeks, the mice were sacrificed, and individual tumours were removed and weighed.

For the in vivo tumour metastasis assay, 200 μl of suspended cells was injected into each nude mouse through the tail vein. After 6 weeks, the mice were sacrificed, and lungs were obtained for further analysis.

### Immunohistochemistry

The tumour and lung tissues were fixed in 4% paraformaldehyde solution at 4 °C for 48 h, embedded in paraffin and sectioned into 4 μm thick slices. Slides were deparaffinized through a series of xylene and graded alcohols. For antigen retrieval, slides were heated in citrate buffer in sub-boiling temperature for 10 min. Then, endogenous peroxidase activity was blocked by 3% H_2_O_2_ for 15 min, and the slides were incubated with primary antibody overnight at 4 °C. Next, the secondary antibody (Gene Tech, Shanghai, China) was applied for 30 min at room temperature. Following DAB staining for the appropriate time, haematoxylin staining was performed. The antibodies against Ki-67 and vimentin were purchased from CST.

### Statistical analysis

Student’s t-test or one-way ANOVA were used for statistical analysis when appropriate. All statistical analyses were performed by SPSS 23.0 software (IBM, Armonk, NY, USA) and were presented as the mean ± standard deviation (SD). All tests were two sided, and *P* < 0.05 was considered statistically significant.
